# Substance Use Disorder in the COVID-19 Pandemic: A Systematic Review of Vulnerabilities and Complications

**DOI:** 10.3390/ph13070155

**Published:** 2020-07-18

**Authors:** Yufeng Wei, Rameen Shah

**Affiliations:** Department of Chemistry, New Jersey City University, Jersey City, NJ 07305, USA; rshah4@njcu.edu

**Keywords:** COVID-19, SARS-CoV-2, coronavirus, substance use disorder (SUD), immunology, neuroinflammation, blood-brain barrier (BBB), hypothalamic–pituitary–adrenal (HPA) axis

## Abstract

As the world endures the coronavirus disease 2019 (COVID-19) pandemic, the conditions of 35 million vulnerable individuals struggling with substance use disorders (SUDs) worldwide have not received sufficient attention for their special health and medical needs. Many of these individuals are complicated by underlying health conditions, such as cardiovascular and lung diseases and undermined immune systems. During the pandemic, access to the healthcare systems and support groups is greatly diminished. Current research on COVID-19 has not addressed the unique challenges facing individuals with SUDs, including the heightened vulnerability and susceptibility to the disease. In this systematic review, we will discuss the pathogenesis and pathology of COVID-19, and highlight potential risk factors and complications to these individuals. We will also provide insights and considerations for COVID-19 treatment and prevention in patients with SUDs.

## 1. Introduction

The novel coronavirus 2019, now officially termed as SARS-CoV-2, causes the coronavirus disease 2019 (COVID-19) by infecting the respiratory system [1]. The disease was first detected and reported in Wuhan, China, in December 2019 [2], and has now spread to over 150 countries on all continents except Antarctica. According to the World Health Organization (WHO) Coronavirus Disease Dashboard, the global tally of coronavirus cases has approached 13.5 million, with a death toll of over 580,000 [3] at the time of writing (15 July 2020). The United States alone has counted over 3.4 million infections and over 131,000 deaths, contributing more than a quarter of both overall infections and deaths globally (John Hopkins University Coronavirus Resource Center) [4]. The WHO characterized the COVID-19 as a pandemic on 11 March 2020 [5]. The fatality rate of the disease is particularly high among patients who are older and who have underlying health issues, such as cancer, diabetes, and compromised lung function or lung disease.

The United Nations (UN) reported that 35 million people worldwide suffer from substance use disorders (SUDs) [6]. In the U.S., the number of individuals experiencing SUDs is 20.3 million [7]. A large number of individuals with SUDs have underlying health conditions, particularly cardiovascular and lung diseases and hepatitis C or HIV-1 infections. Together with complicated socioeconomic issues, these populations are particularly vulnerable to COVID-19 [8]. Despite the fact that researchers and clinicians around the world have collected and disseminated tremendous amounts of data on COVID-19, we have very little knowledge of the interactions and comorbidity of COVID-19 and SUD. In this review, we will analyze relevant COVID-19 and SUD literatures, and highlight the susceptibilities and vulnerabilities for individuals with SUD.

SARS-CoV-2 is known to attack the respiratory tract and could lead to severe lung damage or pulmonary fibrosis [2]. Smokers of tobacco and marijuana, and possibly people who vape [9], are susceptible to chronic obstructive pulmonary disease (COPD) [10,11], which could cause severe complications of COVID-19 and lead to a higher fatality rate [12,13]. Other substances of abuse, such as opioids and methamphetamine, function increasingly through the brain and immune system to indirectly affect the respiratory system. Opioid use disorder (OUD) tends to slow breath rate and decrease blood oxygen content (hypoxemia). An extended period of hypoxemia is one of the major causes of overdose fatality. COVID-19, which shrinks lung capacity, could heighten the condition caused by opioid overdose [14,15]. Methamphetamine may contribute to pulmonary hypertension and edema through cardiomyopathy and restricted blood circulation [16]. Many substances of abuse also impair the bidirectional interactions between the brain and immune responses, resulting in an increase in the infection rates among individuals with SUDs. Some drugs exert proinflammatory effects in the central nervous system (CNS), leading to neuroinflammation. The buildup of proinflammatory cytokines and chemokines in the CNS may exaggerate the already excessive inflammatory response in the peripheral tissues of COVID-19 patients.

In addition to pathological risks that patients with SUDs are facing, highly risky behaviors can put them into even greater jeopardy in the pandemic. Suicide mortality associated with SUDs is significantly higher compared to the general population across all categories, including age, gender, income, and education, and the relative risk of suicide is more prominent in women. People with multiple alcohol, drug, and tobacco use disorders are at a particularly heightened risk of suicide [17]. In a position paper, the International Society of Addiction Medicine (ISAM) Practice and Policy Interest Group noted that people with SUDs suffer from serious health complications, including chronical infections, weakened immune systems, various respiratory, cardiovascular, and metabolic disorders, and a range of psychiatric comorbidities. While they are stigmatized and marginalized with limited access to healthcare, the difference in perceived danger and risk-taking behaviors may put people with SUDs at a higher rate of mortality [18]. Due to the lack of research on SUDs and COVID-19, the group also put together recommendations for health service providers and policymakers regarding the comorbidity of COVID-19 infection and SUDs [18].

## 2. SARS-CoV-2 Infection and COVID-19 Pathogenesis

Coronaviruses are named for their crown-like spikes protruding from the surface of the virion, and can be classified into four genera, known as α, β, γ, and δ. There are seven known coronaviruses that can infect humans. Four of them, α-coronaviruses 2229E and NL63, and β-coronaviruses OC63 and HKU1, infect people on a regular basis and cause common cold symptoms. The other three, all belonging to the β-coronavirus subfamily, are believed to have originated from bats and evolved through jumping animal species to become new human coronaviruses [19,20]. These include the SARS-CoV, the coronavirus that caused severe acute respiratory syndrome (SARS) outbreak in 2003, the MERS-CoV, the coronavirus that caused Middle East Respiratory Syndrome (MERS) outbreak in 2012, and the SARS-CoV-2, the novel coronavirus that is causing the current COVID-19 pandemic railing the whole world.

**Figure 1:**
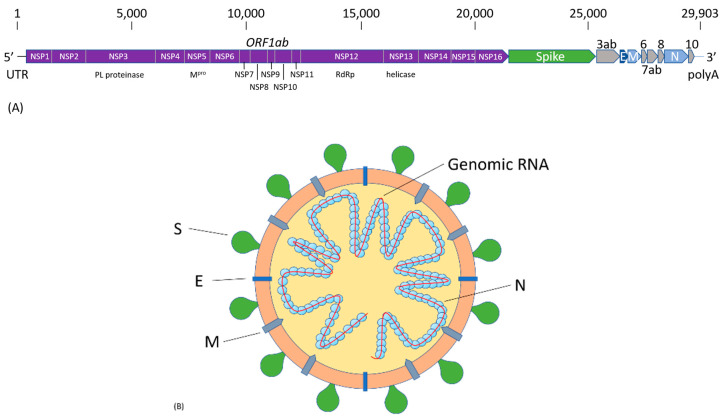
(**A**) Architecture of SARS-CoV-2 genome. The ORF1ab will be translated into two overlapping polyproteins, PP1a, consisting of NSP1-11, and PP1ab, consisting of NSP1-16, with the exception of NSP11, which is part of NSP12 in PP1ab. The rest of the ORFs encode the four structural proteins, S, E, M, and N, and several accessory proteins with unknown functions. (**B**) Structure of SARS-CoV-2 virion. The lipid bilayer. embedded with S, E, and M proteins, capsulizes the single-stranded genomic RNA, which is stabilized by the N protein. The S protein is responsible for the recognition of host cell ACE2 receptor to gain cell entry.

Like all other coronaviruses, SARS-CoV-2 contains a single-strand (ss) RNA genome. The sequence of the 29,903 nucleotides (nt) long SARS-CoV-2 genome was first reported by Chinese scientists and made publicly available on GenBank with accession number MN908947 [21]. The genome organization of SARS-CoV-2 is similar to that of other representative β-coronaviruses. As illustrated in Figure 1a, it is comprised of a 5′-untranslated region (UTR), and at least ten open reading frames (ORFs) that encode non-structural and structural viral proteins, followed by the polyA tail at the 3′-end. The first ORF is the 21,291-nt replicase gene, *ORF1ab*, encoding 16 non-structural proteins (NSP1-16). The subsequent ORFs encode four structural proteins, spike (S), envelope (E), membrane (M), and nucleocapsid (N), as well as several accessory proteins that do not participate in viral replication and transcription. Structural proteins are important in maintaining viral structural and genomic stability. An illustration of SARS-CoV-2 viral structure is shown in Figure 1b. The S protein is responsible for infection and transmission. Non-structural proteins, such as the RNA-dependent RNA polymerase (NSP12), have functions in viral genome transcription and replication. The SARS-CoV-2 genome was found to have 79.6% sequence similarity to SARS-CoV, and shares 96% identity at the whole-genome level to a bat coronavirus (Bat CoV RaTG13) detected in *Rhinolophus affinis* [20].

Similar to SARS-CoV, SARS-CoV-2 recognizes the angiotensin converting enzyme 2 (ACE2) receptor by its S protein and utilizes it for cell entry [20,22]. The heavily glycosylated S protein triggers virus cell entry by fusing the receptor binding domain (RBD) on the S1 subunit to the host ACE2 receptor, engaging the transition of S2 subunit to a stable post-fusion conformation [23]. Cryo-electron microscopy (EM) structures of the pre-fusion [23] and post-fusion structures [24] of the S protein have been reported. The SARS-CoV-2 S protein has been shown to have a much higher binding affinity to the ACE2 than the SARS-CoV S protein [23,25]. The S protein contains 22 N-linked glycans, and the complex glycosylation is likely to play a role in shielding and camouflaging for immune evasion of the virus [26,27]. The S protein is activated by type II transmembrane serine protease (TMPRSS2), a host protease co-expressed with ACE2 on the cell surface [24,28]. In cells not expressing TMPRSS2, other proteases, such as cathepsin B/L, may activate the S protein and facilitate viral entry [29].

Upon cell entry, SARS-CoV-2 has a similar life cycle and pathogenesis as other β-coronaviruses, including SARS-CoV and MERS-CoV [30]. Upon ACE2 receptor binding, the virus fuses its membrane with the host cell plasma membrane, releasing its genomic RNA into the cytoplasm. Since the viral RNA is similar to the human messenger RNA (mRNA), it triggers the host ribosome to start translating the viral RNA and producing viral proteins. The viral replicase ORF is translated into two overlapping polyproteins, PP1a (NSP1-11) and PP1ab (NSP1-16), which require extensive processing. NSP5, the 33.8-kDa main viral protease (M^pro^), also referred to as the 3-chymotrypsin-like protease (3CL^pro^), performs the function by autolytic cleavage of the protease itself, and then subsequently digests the polyproteins into 16 non-structural proteins. NSP12, known as the RNA-dependent RNA polymerase (RdRp), together with NSP7 and NSP8, carries out the critical process of the viral RNA synthesis, and is central to the viral replication and transcription cycle. The N-terminal non-structural protein, NSP1, has been shown to bind to the 40S small ribosomal subunit, shutting down all host cell protein production by blocking the mRNA entry tunnel. NSP1 binding to ribosomes and blocking host cell translation effectively inhibits type-I interferon (IFN-I)-induced innate immune response by turning off the retinoic acid-inducible gene (RIG)-I antiviral sensor [31]. The inhibition of the IFN-I-induced innate immunity allows the assembly of viral particles inside the host cell. The newly produced structural proteins, S, M, and E, are inserted into the endoplasmic reticulum (ER) or Golgi membrane, while the N protein associates with the newly synthesized viral RNA to stabilize the genome. The viral particles are assembled into the ER-Golgi intermediate compartment (ERGIC), fuse with the plasma membrane, and bud off the host cell. The released virions will further infect more cells. The functions of other NSPs are not fully understood. A comparative structural genomics study revealed a possible functional intra-viral and human-virus interaction network of NSPs [32]. Recurrent mutations in the SARS-CoV-2 genome have been identified in some NSPs and the S protein, suggesting ongoing adaptations of the coronavirus through transmission [33]. Particularly, the D614G mutation in the S protein makes it more stable, and the virus becomes more infectious and transmissible [34,35]. This mutated virus is the dominant form in Europe and North America since March 2020 [36].

## 3. Vulnerability of Substance Use Disorders (SUDs) in COVID-19

Underlying medical conditions can put individuals at increased risk for severe illness from COVID-19. The comorbid conditions include COPD, cardiovascular diseases, other chronical respiratory diseases, diabetes, obesity, and cancer. According to a large-scale study with 72,314 cases conducted by the Chinese CDC, case-fatality and mortality rates are significantly increased in patients with comorbid conditions comparing to those with no underlying conditions (Table 1) [12]. In a study in New York City, the epicenter of the COVID-19 pandemic in the U.S., comorbid conditions are highly associated with hospitalization and severity of the illness (Table 2) [37].

A nationwide case-control study in Korea also confirmed that diabetes, hypertension, and chronic respiratory disease, among others, were associated with severe COVID-19 [38]. Individuals with SUDs commonly experience respiratory and cardiovascular disorders, including hypertension and COPD, and have undermined immune systems, making them particularly vulnerable in COVID-19. A significant portion of individuals with SUDs have underlying medical conditions, and are more likely to be marginalized. According to a recent study in British Columbia, Canada, among 19,005 individuals who had one or more non-fatal overdose events between 2015 and 2017, 10,649 (56.0%) had a record of receiving social assistance, and 5716 (30.0%) had no fixed address record. These individuals with a history of overdose are more likely to have at least three known chronical conditions associated with COVID-19 severity, including chronical pulmonary disease, diabetes, and coronary heart disease, with adjusted odds ratios (ORs) to be 2.01, 1.24 and 2.08, respectively, with reference to people without an overdose [39]. During the COVID-19 pandemic, risks of abusing substances and addictive behaviors are also increasing. The stress and social isolation associated with the response to COVID-19 increases the risk of alcohol abuse and misuse, which is known to suppress immune systems and cause emotional dysregulation [40]. A study in China showed that relapses of alcohol and smoking abuse were prominent (18.7% and 25.3%, respectively), and 32.1% of regular drinkers and 19.7% of regular smokers increased alcohol and cigarette consumption [41].

SARS-CoV-2 can attack and damage human organs through two major events: direct viral attacks against target organs and abnormal immune responses and inflammation [42]. Initial evidence focuses on the damage to the respiratory system and the lung, and is correlated with clinical symptoms of the patients [2,12,20,21]. The identified viral entry receptor, ACE2, is abundantly expressed in the epithelial cells along the respiratory tract and the lung alveoli [29,43,44]. High level expression of ACE2 receptors is also reported in organs and tissues outside the respiratory system, including the heart, kidney, and intestine [45]. Therefore, these organs are the potential targets of and could be damaged by SARS-CoV-2. As mentioned earlier, smoking of tobacco and marijuana directly impairs respiratory system. Other substances of abuse can cause cardiovascular diseases, which amplify respiratory and pulmonary complications. We will discuss these complications in the next section.

**Figure 2:**
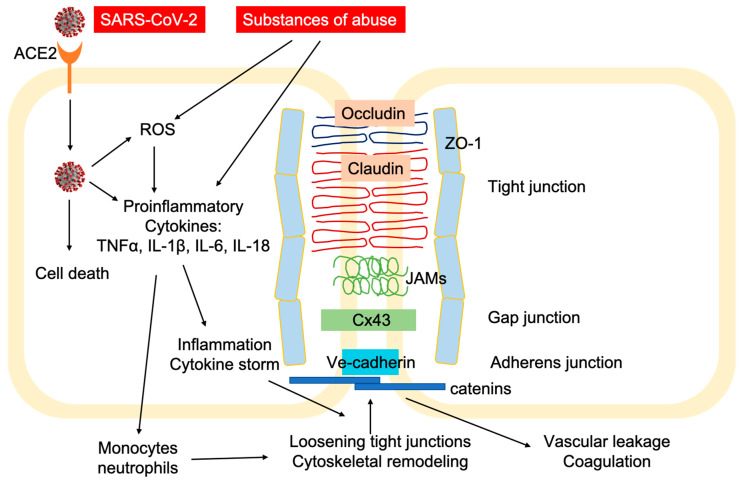
Schematic illustration of vascular endothelial junctional architecture. SARS-CoV-2 infects vascular endothelial cells through the surface-expressed ACE2 receptor. The internalization of the virus can cause endothelial cell death, reactive oxidative species (ROS), and the release of various proinflammatory cytokines. Excessive inflammation, and potentially cytokine storm, induces the loosening of the tight junction complex and cytoskeletal remodeling, leading to vascular leakage and coagulation. Various substances of abuse exert similar effects at the brain endothelial junctions, disrupting the BBB and allowing viral infection in the CNS.

More severely, ACE2 is abundantly expressed in vascular endothelial cells [45]. Several clinical cases have been reported to indicate direct involvement of vascular endothelial cells in COVID-19 pathology at different organs, suggesting that the damages to the lung, heart, kidney, liver, small intestine, and bowel, are actually caused by endotheliitis (endothelialitis). Direct viral infection of endothelial cells induces inflammation and inflammatory cell death at the endothelium (Figure 2) [46]. Comparing lung tissues from deceased patients of acute respiratory distress syndrome (ARDS) associated with influenza and COVID-19, the lungs from COVID-19 patients displayed distinctive vascular impairments of the pulmonary vessels. Most significantly, viruses were found inside the endothelial cells of the lung tissues from COVID-19 patients, which disrupted cell membrane, caused prevalent thrombosis with microangiopathy, and induced elevated intussusceptive angiogenesis [47]. A greater number of ACE2+ endothelial cells were found in COVID-19 patients, correlating to changes in endothelial morphology, including disruption of endothelial cell junctions, cell swelling, and detachment from the basal membrane. The presence of the SARS-CoV-2 virus inside the endothelial cells, together with the induced inflammation, may directly contribute to the endothelial injury [46,47]. Although there are no reported cases yet, one potential target of SARS-CoV-2 infection is the brain microvascular endothelial cell (BMVEC). BMVECs line up the microcapillary beds and form the blood-brain barrier (BBB) together with brain astrocytes and pericytes. The BBB prevents pathogens and toxins from trespassing into the brain side. Tight-junction (TJ) protein complexes, composed of occludin, claudins, junctional adhesion molecules (JAMs) and membrane-directed scaffolding protein zonulae occludentes (ZO), form a physical inter-endothelial barrier that strictly controls migration of molecular and cellular contents from the circulation into the brain (Figure 2) [48,49]. High expression of efflux pumps and stereospecific solute transporters at the endothelium additionally limits molecules from crossing the barrier [50,51,52]. The BBB plays an essential role in protecting the brain from pathogen invasion. Viral infection of BMVECs could result in endothelial dysfunction, leakage, and even rupture, and is detrimental to the BBB integrity. The damaged BBB allows the virus to migrate into the brain side, and infect neuronal tissues [53]. Another possible route of CNS invasion could be through invading the peripheral nerve terminals and then entering the CNS via trans-synaptic transfer [54]. The first case of meningitis associated with SARS-CoV-2 has been reported, in which viral RNA was detected in the cerebrospinal fluid (CSF) of the patient [55]. Clinical evidence from Wuhan, China showed that more than 1/3 of COVID-19 patients had neurological symptoms, and CNS involvement was linked to the prognosis and severity of the disease [56,57]. Substances of abuse have been shown to severely compromise the endothelial barrier at the BBB, leading to increased BBB permeability and possibly intensified brain damage in COVID-19 (Figure 2).

The other aspect of COVID-19 pathology involves abnormal immune responses, which could exaggerate into a cytokine storm [58,59]. SARS-CoV-2 dramatically promotes host cell kinase activities, including casein kinase II (CK2) and p38, and stimulates production of diverse cytokines [60]. Evidence has shown that COVID-19 elevates proinflammatory cytokines and chemokines, including tumor necrosis factor (TNF)-α, interleukin (IL)-1β and IL-6, granulocyte-colony stimulating factor (G-CSF), interferon γ (IFN-γ)-induced protein-10 (IP-10), monocyte chemoattractant protein-1 (MCP-1), and macrophage inflammatory proteins-1α (MIP-1α) [61,62,63]. Although there has been no reported evidence, it is possible that pattern recognition receptors, such as toll-like receptors (TLR3, TLR7, and TLR8), RIG-I, and nucleotide-binding oligomerization domain (NOD)-like receptors (NLRP1, NLRP3, and NLRP12), are also activated by COVID-19 through innate immunity [64]. It has been reported that IL-6 was significantly increased in severe COVID-19 cases, and its level was closely correlated with the severity of patients [65]. Human bronchial epithelial cells infected with SARS-CoV-2 showed elevated expression of type I and type III IFNs and IL-6 [44]. Furthermore, type I interferon, IFN-α, stimulates the expression of ACE2, the molecular target of SARS-CoV-2, in primary human nasal epithelial cells [43]. Type III interferon, IFN-λ, has been shown to disrupt the lung epithelial barrier by direct inhibition of lung epithelial proliferation and repair, contributing to COVID-19 pathogenesis in the lower airways [66]. The upregulation of IL-6 and other proinflammatory cytokines was also observed in SARS cases [67] and influenza infection [68]. Substances of abuse can induce high level of expression of proinflammatory cytokines and chemokines in the CNS and cause neuroinflammation, which can worsen inflammatory responses in COVID-19. Details will be discussed in the next section.

**Figure 3:**
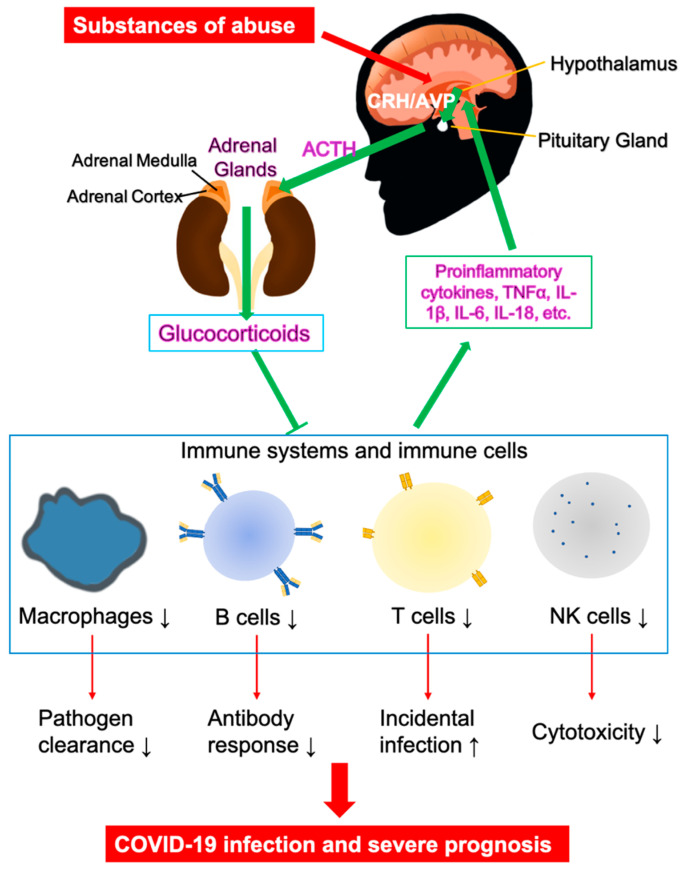
Bidirectional communication between the brain and the immune system. The HPA axis: upon activation (cytokines, pathogens, etc.), the hypothalamus in the brain produces CRH and AVP, activating anterior pituitary, which secretes ACTH. ACTH circulates with general blood stream to reach adrenal gland, which synthesizes the anti-inflammatory molecule, glucocorticoids. Glucocorticoids suppress the immune system and the expression of proinflammatory cytokines, which concludes the negative feedback and turns off the HPA axis. Glucocorticoids suppress the activities of various immune cells, including macrophages, dendritic cells, and T cells, which are responsible for cytokine release. The immunosuppression also involves inhibition of NK cells, B cells, and T cells for reduced cytotoxicity, antibody production, and T cell-mediated immune responses. Substances of abuse alter the HPA axis. Excessive production of glucocorticoids suppresses immune responses to viral infection, leading to high incidences of infection and severe infection in COVID-19. Arrows indicate stimulation; blunted arrows indicate inhibition.

The bidirectional communication between the brain and the immune system plays a critical role in COVID-19 pathogenesis. It has been well established that brain-immune interactions are widespread and significant. For instance, the immune system produces hormones and neurotransmitters [69,70,71], while the anterior pituitary cells in CNS produce proinflammatory cytokines, such as IL-6 [72]. Microglial cells are immune effectors in the CNS, which produce and secrete cytokines and neurotrophic or neuron survival factors upon inflammation and injury [73]. For pathogen infections, the innate immunity provides the first line of defense through recognition of pathogen-associated molecular patterns (PAMPs), initiating nonspecific cellular and humoral responses and rapidly activating nonspecific neural responses, including systemic hormonal responses through the hypothalamic-pituitary-adrenal (HPA) axis (Figure 3) [74]. Consisting of the hypothalamus of the brain, and endocrine organs, the pituitary and cortex of the adrenal glands, the HPA axis is responsible for systematic inflammation control. The paraventricular nucleus (PVN) of the hypothalamus plays the main governing role of the HPA axis, releasing a wide range of neuropeptides, including the corticotrophin-releasing hormone (CRH) and arginin-vasopressin (AVP). These neuropeptides reach the anterior pituitary (AP) to activate corticotrope cells to secrete adrenocorticotropic hormone (ACTH). ACTH subsequently enables the synthesis and secretion of glucocorticoids in the zona fasciculata of the adrenal cortex through melanocortin type 2 receptors [75]. The physiological feedback loop involves releasing immune mediators and cytokines, such as IL-1, IL-6, and tumor-necrosis factors (TNFs), by the innate immune system to activate neural responses, which amplify local inflammation to contain and eliminate pathogen invasions. Upon pathogen clearance, the brain responds by activating the HPA axis and releasing anti-inflammatory molecules, glucocorticoids, from the adrenal cortex. The release of this final product of the HPA axis sends a signal to the immune system to terminate the inflammatory responses, completing the hormonal negative-feedback loop. Glucocorticoids also negatively regulate the HPA axis itself, restoring host homeostasis, including CNS and cardiovascular system, as well as metabolic balances. The interplay between the nervous system and the immune system plays a critical role in forming a cohesive and integrated early host response for pathogen clearance through an optimized innate inflammatory response. Impairment of the HPA axis by various substances of abuse could render the host highly susceptible to inflammation and even increased mortality from septic shock from exposure to infectious and proinflammatory triggers, including COVID-19. Inappropriate and excessive CNS responses could predispose the host to extreme inflammation, including cytokine storm that has been observed clinically in influenza [68,76], SARS [77], and COVID-19 [59]. Excessive activation of the HPA axis and the release of glucocorticoids by several substances of abuse will suppress the activities of various immune cells, including macrophages, dendritic cells, and T cells, and will inhibit activities of NK cells, B cells, and T cells (Figure 3) [74]. The immunosuppression reduces antibody production, cytotoxicity, and T cell-mediated immune responses, and is linked to higher incidences of pathogen infections, slowed recovery, and severe disease progression in COVID-19.

## 4. Effects of Commonly Abused Substances on COVID-19

For individuals with SUDs, both the infection of vascular endothelial cells and the proinflammatory immune responses of COVID-19 could pose severe risks. It has been well studied that substances of abuse can cause irreversible BBB damage [78] and impair the HPA axis and immune responses [75]. To understand the vulnerabilities of SUDs in COVID-19 infection at the molecular level, we will examine the brain-immune interactions and the HPA axis attenuation, neuroinflammation, immunosuppression, and BBB impairment induced by most commonly abused substances in the comorbidity of COVID-19.

### 4.1. Nicotine

Nicotine is available in the form of tobacco, which also contains many other types of chemicals. Tobacco smoking is associated with arterial thrombosis and atherosclerosis in the heart, abdomen, and neck [79], and is associated with increased risk of stroke, pulmonary disease, and emphysema [80]. As nicotine damages the lung directly, smoking is one of the leading causes of COPD. Smokers and COPD patients are of high risk in developing severe disease and have a higher mortality rate in COVID-19 [13]. Smoking can significantly worsen COVID-19 progression, with more critical conditions and higher fatality [81]. Cigarette smoking increases the number of alveolar macrophages (AMs), the innate immune cells in the lung by several fold. These cells secrete lysosomal enzyme, elastase, which can damage connective tissue and parenchymal cells of the lung, one of the contributors to the COPD pathogenesis [82].

Nicotine is also an important immune modulator. It significantly reduces antibody responses and T-cell proliferation [83]. The immune suppression by nicotine, particularly the decrease in CD8+ T-cells that facilitate the rapid resolution of acute viral infections, increases the susceptibility of smokers for viral infections [84]. Analysis of clinical data has indicated that smokers are twice as much as non-smokers to contract the virus, have a more severe disease progression, and have higher mortality rates [85].

Nicotine induces BBB leakage and increases BBB permeability by diminishing the expression of tight junction proteins, including occludin, claudin-3, ZO-1, and JAMs [86,87,88,89]. Nicotine alters actin cytoskeleton arrangement in the BMVEC, which also greatly increases BBB permeability, resulting in a surge of bacterial invasion to the brain [90]. Nicotine induces oxidative stress, which can progressively compromise the BBB integrity [91]. Nicotine increases gene expression of proinflammatory cytokines, TNFα, IL-1β, and IL-18, and chemokines, CCL2, CCL8, and CXC3CL1, and suppresses anti-inflammatory factors, Bcl6, IL-10, and CCL25 in the brain microvessels [92]. Nicotine’s detrimental effects on the BBB and induced neuroinflammation are serious concerns for COVID-19.

### 4.2. Alcohol

The history of fermentation production and the use of alcohol can be dated back to 10,000 BC. Although light-to-moderate consumption may arguably have positive health benefits, particularly in lowering cardiovascular risks [93,94], high dose alcohol can have severe neurotoxic effects and cause dementia [95]. Alcohol has been linked to liver damage, inflammation of the pancreas and stomach, and neurodegenerative disorders. High alcohol consumption changes gene expression of immune response genes in the brain region frontal cortex [96]. Proinflammatory signaling is also connected to high alcohol intake [97]. Binge alcohol (blood alcohol content B.A.C ≥ 0.08%) elevates expression of proinflammatory cytokines, IL-1β and IL-6, and chemokine CCL-2 (MCP-1) [98]. Binge alcohol can also damage various organs, including the gut, liver, and brain [99], and lead to spleen atrophy in a hippocampus-mediated fashion [100]. As suggested in a comparative risk assessment using the margin of exposure (MOE) benchmark, alcohol is considered to have the highest risk of mortality [101]. The alcohol-induced inflammatory cytokine release could expose COVID-19 patients to excessive inflammatory responses. The spleen damage by alcohol could weaken the immune response in COVID-19 by reducing the production of antibodies and lymphocytes against the virus [102].

Alcohol can induce BBB disruption by decreasing the expression of tight junction proteins and increasing mitogen-activated protein kinase (MAPK) activities [103]. Alcohol may also stimulate inositol 1,4,5-triphosphate receptor (IP3R)-operated intracellular Ca^2+^ release, activating myosin light chain kinase (MLCK). The heightened kinase activities lead to the phosphorylation of cytoskeletal and tight junction proteins, compromising the BBB integrity [104]. Alcohol-mediated oxidative stress in BMVEC can also activate MLCK that alters cytoskeleton and tight junction protein structures, causing BBB leakage [105]. Disruption of the BBB associated with chronic alcohol use could increase the possibilities of invading pathogens, including SARS-CoV-2, to infiltrate the brain.

### 4.3. Marijuana

Marijuana, also known as cannabis, is the most frequently used illicit substance of abuse in the U.S. Currently, 11 states and the District of Columbia have legalized the recreational use of marijuana, and 16 states have decriminalized marijuana use and possession. The use of marijuana for medical purposes is legal in 33 states. It is, however, illegal under federal law to use and possess marijuana. It is classified as a Schedule I substance by the US Drug Enforcement Administration (DEA), indicating a high potential for abuse and no accepted medical use, despite the fact that medical marijuana has been well established [106,107]. Two cannabinoids, ∆^9^-tetrahydrocannabinol (THC) and cannabidiol (CBD), are the main active ingredients in marijuana. Cannabinoids act on the endocannabinoid system (ECS), which is composed of two cannabinoid receptors, CB1, expressed in most neuronal cells, and CB2, expressed predominantly in immune cells [108]. THC is the component that produces psychotropic effects through stimulating the CB1 receptor and mediating the inhibition of neurotransmitter release [109]. CBD is considered to regulate immune response, such as cytokine release, and blood pressure, with little to no psychotropic side effects, as CBD only binds to CB2 but not to CB1 receptors [109]. THC can also activate CB1 receptor in the cardiovascular system, which has been associated with adverse cardiovascular events, including myocardial infarction, cardiomyopathy, arrhythmias, stroke, and cardiac arrest [110].

Both THC and CBD are lipophilic, allowing them to readily pass through the BBB and enter the CNS. THC is an immunosuppressor. It has been reported to suppress antibody response and T lymphocytes activities. It prevents macrophage and macrophage-like cells, such as microglia, from migrating towards the nodes of microbial invasions. It also suppresses proinflammatory factors and promotes anti-inflammatory activities [111]. THC downregulates proinflammatory cytokines, IL-1α, IL-1β, and TNFα. By dampening immune responses to invading pathogens, THC could make the host more susceptible to viral infections, such as HIV-1 [111] and possibly COVID-19. Other harmful effects of marijuana include cardiovascular, cerebrovascular, and neurological complications, such as stroke, cognitive dysfunction, and behavioral problems [112]. Some evidence suggested a link between smoking marijuana and risk of COPD [113], one of the major risk factors for COVID-19 complications, but the risk is only significant if the individuals also smoked tobacco [114]. On the contrary, a study, which has not been peer-reviewed at the time of preparation of this article, showed the beneficial effect of CBD in preventing COVID-19 by modulating ACE2 expression and downregulating serine protease TMPRSS2 [115].

### 4.4. Opioids

Opioids, including illegal drug heroin, synthetic drug fentanyl, prescription pain-killing drugs oxycodone (OxyContin^®^), hydrocodone (Vicodin^®^), codeine, and morphine, are effectors on the endocrine system [116]. They act as immune suppressors that impair the function of macrophages, natural killer (NK) cells, and T-cells, and are associated with higher risks of infectious diseases, such as pneumonia [117]. The endogenous opioid system (EOS), comprising three naturally occurring opioid peptides (β-endorphins, dynorphins, and enkephalins) and three classes of opioid receptors, µ (MOR), δ (DOR), and ĸ (KOR), are tightly linked to substance abuse and the development of addiction, and are responsible for systemic infection [118]. Activation of opioid receptors in the brain stem could lead to respiratory depression and overdose fatality [119]. Respiratory depression is a leading factor to hypoxemia in COVID-19 complications. Morphine desensitizes the HPA axis and inhibits the release of anti-inflammatory glucocorticoids through potentiating proinflammatory cytokine, IL-1β [120]. Inhibition of endogenous glucocorticoid and activation of IL-1β by opioids could significantly increase the severity and inflammatory response of COVID-19 in patients with OUD. It has been shown that individuals with OUD are more susceptible to opportunistic infections [121], disposing these individuals at a higher chance of contracting the virus in COVID-19.

### 4.5. Cocaine

Cocaine is a potent stimulant, and is the second most abused illicit drug after marijuana. Cocaine can be abused in several forms, such as chewing the leaves of *Erythroxylum coca* tree, injecting the water-soluble hydrochloride salt form, and smoking or snorting pure, freebase form, called “crack”. One of the most significant pathophysiological effects of cocaine is its cardiotoxicity, which has been well documented and extensively reviewed [122,123,124,125,126]. Cardiac arrhythmias and acute myocardial ischemia or infarction (MI) are the leading causes of cocaine-induced sudden cardiac death. Other cardiovascular diseases associated with cocaine include heart failure, cardiomyopathies, aortic dissection, and endocarditis. Cocaine blocks voltage-dependent Na^+^ and K^+^ channels in the sinoatrial node and the myocardium, depressing cardiovascular contractility [127]. Due to its function as an ion channel blocker, cocaine has been effectively used as a local anesthetic [128]. Cocaine can also induce binding and opening of L-type Ca^2+^ channels, causing the influx of Ca^2+^ in cardiomyocytes and elevation of intracellular Ca^2+^ concentration. This second messenger pathway may also lead to cardiac arrhythmia [129]. Clinically, cocaine increases myocardial oxygen demand by increasing heart rate and hypertension, while it decreases the oxygen supply due to coronary vasoconstriction [130,131,132]. Cocaine impairs endothelial functions [133], sensitizes constrictor effects of catecholamines [134], and causes microvascular diseases and thrombosis [135,136]. The cocaine-mediated oxygen imbalance can be particularly detrimental in COVID-19, in which the coronavirus can cause hypoxemia because of the diminishing of lung capacity.

Cocaine exerts its effect through binding to three monoamine transporters on nerve terminals: the serotonin transporter (SERT), the dopamine transporter (DAT), and the norepinephrine transporter (NET), with *K*_i_ of 0.14, 0.64, and 1.6 µM, respectively [137]. Upon binding to these transporters, cocaine inhibits the reuptake of the neurotransmitters from the synaptic cleft, leading to prolonged synapses and activation of postsynaptic receptors. Cocaine also binds directly to two classes of neurotransmitter receptors, muscarinic acetylcholine and sigma receptors [138,139,140]. The interactions with the transporters and the receptors form the molecular basis for cocaine neurotoxicity. Cocaine stimulates the HPA axis, increasing the secretion of neuronal peptide CRH, which leads to subsequent releasing of β-endorphin and ACTH. Through general circulation, ACTH reaches the adrenal glands and promotes the biosynthesis of glucocorticoids [141]. Cocaine-induced stimulation of the HPA axis and immune suppression can alter antibody formation, lymphocyte subset profile, and lymphocyte proliferation. Cocaine suppresses responses to the proinflammatory cytokine, IL-6, and dampens cytotoxic activation of macrophages, natural killer cells, and cytotoxic T lymphocytes [142]. Due to compromised immune responses, cocaine abusers have considerably high incidences of viral infections, including human immunodeficiency virus (HIV), influenza, and potentially SARS-CoV-2.

Cocaine can induce BBB dysfunction, disrupt neurovascular capillaries and basement membrane [143], and increase BBB permeability [144,145,146]. The detrimental effects of cocaine on the BBB are partially attributed to the loss of tight junction protein complexes, including ZO-1 and JAM-2 [146,147,148,149]. Cocaine also increases the expression of matrix metallopeptidase (MMP)-1, which contributes to the rearrangement of the cytoskeleton structure of the basement membrane [147,148]. The adverse effects of decreased tight junction protein complexes and remodeling of the basement membrane fibers cause the BBB leakage and make it open to peripheral toxins and viruses, including SARS-CoV-2. An increase in proinflammatory cytokine, TNFα, has also been reported in BMVECs exposed to cocaine [150], which could be a concern for endothelial health in COVID-19.

### 4.6. Amphetamine, Methamphetamine (METH), and 3,4-Methylenedioxymethamphetamine (MDMA, Ecstasy)

d-Amphetamine and its synthetic derivatives, METH and MDMA, are addictive psychostimulants associated with neuropsychiatric complications, including deficits in attention, memory, and executive functioning [151,152]. Amphetamines mediate neurodegenerative changes in the brain, including persistent loss of dopamine (DA) transporters [153,154,155,156,157] and receptors [158], loss of serotonin (5-HT) transporters [159], and decrease in dopamine and serotonin level and its metabolites [160,161,162]. METH has been linked to various cardiac pathologies, including hypertension, tachycardia, and congestive heart failure or cardiomyopathy [16]. Clinically, METH abusers commonly showed dilated and poorly contracting left ventricles (LV) and substantially lowered left ventricular ejection fractions (LVEF) in comparison to non-users [163]. METH-associated cardiac pathologies are a major cause of pulmonary edema. The reduced lung capacity due to the fluid collection in the lungs and constriction of blood vessels can severely complicate COVID-19 symptoms and negative prognosis.

Amphetamines stimulate the HPA axis and increase the plasma glucocorticoids through a CRH-dependent mechanism involving serotonin [164,165,166,167]. The stimulation increases the production of CRH and AVP in the PVN neurons, which, in turn, activates the production of ACTH in corticotropic cells in the anterior pituitary gland. ACTH circulates through the systemic blood stream to reach and activate the adrenal cortex to release glucocorticoids. The HPA axis is an essential component of the response to pathogen infections. However, chronic activation of the HPA axis and markedly increased glucocorticoid due to the recreational use of amphetamine, METH, and MDMA can be harmful to the brain. Amphetamines modify brain expression of the genes and proteins associated with the HPA axis, including the glucocorticoid receptor (GR) and the mineralocorticoid receptor (MR). The remodeling of brain cells and disruption of the HPA axis are the hallmarks of depression and anxiety/despair states associated with drug use [168,169]. As mentioned earlier, the disruption of the HPA axis and the associated immune suppression could pose the drug abusers to a higher risk of viral infections. SARS-CoV-2, known for its high infection rate, will be particularly harmful to individuals with defective immune systems related to the use of addictive drugs.

Amphetamine-like psychostimulants can trigger inflammatory processes, compromise neurogenesis in the brain, and damage the BBB integrity [170]. METH, for example, is strongly associated with ischemic stroke and hypoxia [171,172]. Binge use of METH causes a sustained reduction in global and cerebral blood flow [173,174]. METH is also known to damage the central nervous system (CNS) by compromising the integrity of the blood-brain barrier (BBB) [175,176,177]. METH and MDMA can decrease the expression of tight junction proteins, including ZO-1, occludin, and claudin-5. These drugs activate microglia and astrocyte to secrete proinflammatory cytokines and chemokines, as well as vasoactive factors, and elevate expression of peptidases, such as MMP-1 and MMP-9, to degrade tight junction proteins and modify BBB basement membrane structure [178,179]. METH abuse has been shown to increase brain infection of peripheral bacteria and viruses [180,181,182]. Similarly, MDMA has been shown to cause BBB dysfunction with increased BBB permeability [183], and to excessively activate astrocytes and microglia [184]. It may lead to edema [183]. With BBB dysfunction, it is almost certain that the risks of SARS-CoV-2 invasion into the brain will be immensely heightened among individuals using or abusing these psychostimulants, and complications in these patients are vastly expected.

Neuroinflammation induced by METH and MDMA also plays a major role in BBB damage and may deteriorate COVID-19 conditions. COVID-19 patients exhibit abnormal immune responses related to high levels of proinflammatory cytokines, including TNFα and IL-6. METH significantly increases the expression of TNFα and IL-6 in the hippocampus, frontal cortex, and striatum [185]. The expression of these proinflammatory cytokines is linked to METH-induced microglial activation [186,187]. MDMA also elevates the expression of proinflammatory cytokines, such as IL-1β, in the brain [188]. The excessive expression of proinflammatory cytokines in brain tissues could further damage the BBB and cause oxidative stress [78,189]. Neuroinflammation poses a major risk for individuals with COVID-19.

### 4.7. Summary of Roles of Substances of Abuse in COVID-19

**Figure 4:**
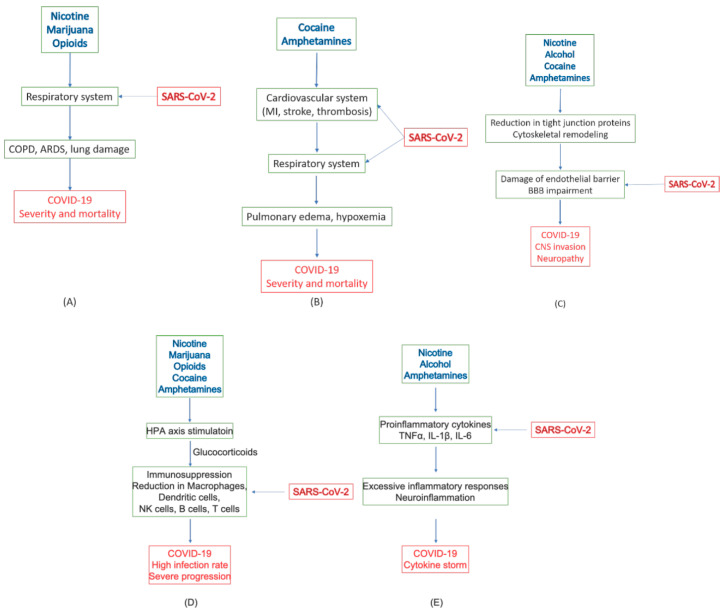
Pathological effects of substances of abuse on various tissues and systems and their implied complications in COVID-19. (**A**) Respiratory system; (**B**) Cardiovascular system; (**C**) Vascular endothelium; (**D**) HPA axis stimulation and immunosuppression; (**E**) Proinflammation and neuroinflammation.

Substances of abuse may lead to COVID-19 complication and severity in several ways. Smoking tobacco and marijuana could cause direct damage to the respiratory system, such as COPD. Other substances mostly work through modulating brain and immune functions, including the promotion of proinflammatory factors, suppression of immune responses, and impairment of the BBB. Neuroinflammation induced by several substances of abuse and inflammatory activities caused by COVID-19 in the peripheral tissues may mutually intensify the adverse effects of one another, leading to negative progression of the disease. With impaired HPA axis and immune imbalance, the patients are highly susceptible to SARS-CoV-2 infections. Compromised BBB may pose a high risk of viral infection in the brain tissue. Figure 4 sketches the possible pathological effects of commonly abused substances on various tissues and systems and their connection to COVID-19 complications. These adverse effects of these substances on the respiratory system, cardiovascular system, the immune system, and the CNS, as well as their relations to the severity and negative prognosis of COVID-19, are summarized in Table 3.

## 5. Strategies of Treatment and Prevention for Individuals with SUDs

### 5.1. General Approaches for Treatments and Vaccines

The therapeutic strategies for COVID-19 have been focused on repurposing existing drugs against this novel coronavirus [190,191,192]. Among these repurposed drugs, the antimalaria drugs chloroquine and hydroxychloroquine [193,194] are extremely controversial, including a retracted study [195] and a terminated solidarity clinical trial by the WHO [196]. The U.S. Food and Drug Administration (FDA) recently revoked its Emergency Use Authorization (EUA) to treat COVID-19 [197]. Other drugs include the anti-HIV drugs lopinavir-ritonavir in combination with ribavirin [198,199,200], tumor chemotherapy drugs, doxorubicin and paclitaxel [201], traditional herbal medicines [202], broad spectrum antiviral drug niclosamide [203], Janus-associated kinase (JAK) 1 and 2 inhibitor, ruxolitinib [204], anti-influenza drug favipiravir [205], antiviral drug remdesivir [206,207,208], and most recently, a commonly used steroid, dexamethasone [209]. Many of these drugs primarily target the RdRp (NSP12) or the main protease, M^pro^. A cryo-EM structure of NSP12 in complex with its cofactors NSP7 and NSP8 has been reported [210]. Remdesivir, the only proven effective drug against COVID-19 so far, is found to potently inhibit RdRp in MERS-CoV [211] and SARS-CoV2 [212]. Ribavirin and Favipiravir also function as RdRp inhibitors. The crystal structure of the M^pro^ with its inhibitor has been recently solved and reported [213,214]. Due to its importance in viral production, and the lack of similar proteins in human cells, the M^pro^ is considered an important drug target in treating COVID-19 [213,215,216]. Niclosamide and the anti-HIV combination drug lopinavir-ritonavir, are inhibitors of the main protease. Structure-based design has led to the development of new inhibitors of the M^pro^ with desirable pharmacokinetic properties and low toxicity [217]. The SARS-CoV-2 entry point, ACE2, and the associated protease, TMPRSS2, are also considered potential drug targets [28].

Tremendous research efforts have been put into identifying, isolating, and developing neutralizing antibodies against SARS-CoV-2. The S protein, which plays a key role in recognizing and binding to the ACE2 receptor to gain entry into the host cell, is the main focus for developing neutralizing antibodies and vaccines against SARS-CoV-2 [24,25]. Neutralizing monoclonal antibodies isolated from convalescent COVID-19 patients were found to block the RBD surface of the S protein from binding to ACE2, and these antibodies showed effectiveness in reducing viral infection in animal models [218,219,220]. The crystal structure of the RBD-bound antibody provided a clear picture on inhibition of viral interaction with ACE2 [221]. The neutralizing antibodies isolated from SARS-CoV patients seemed to cross-neutralize SARS-CoV-2 [222]. The RBD is not the only site that neutralizing antibodies may block. A monoclonal antibody isolated from convalescent COVID-19 patients exhibits high neutralization potency against SARS-CoV-2. This antibody does not bind to the RBD, but it tightly associates with the N-terminal domain of the S protein [223]. A recombinant antibody fused with the human ACE2 extracellular domain displayed desired neutralizing properties in vivo and in mice [224]. Single-domain camelid antibodies from a llama were found to cross-react and neutralize MERS-CoV, SARS-CoV, and SARS-CoV-2 S pseudotyped viruses [225]. Cocktails of antibodies that simultaneously bind to different epitopes of the RBD may provide more potent neutralizing power and significantly reduce virus escaping through mutations [226,227,228]. 

Vaccines are crucial in combating the COVID-19 pandemic. The RBD of the S protein is, again, the main target for vaccine development [229]. According to WHO “Draft landscape of COVID-19 candidate vaccines”, there are 13 candidates currently in clinical trials, and 128 candidates in preclinical stage [230]. These vaccine candidates cover almost all technology platforms, including more traditional non-replicating or replicating viral vector, inactivated or live attenuated virus, recombinant protein subunit, to more recently developed nucleic acid (DNA or RNA), peptide, and viral-like particle [231]. There have been some encouraging results from clinical trials, including the first mRNA vaccine, mRNA-1273 [232,233], and an adenovirus type-5 (Ad-5) vectored vaccine [234].

### 5.2. Challenges for Individuals with SUDs

With the race to therapeutics and vaccines towards COVID-19, rarely have the efforts specifically been directed at SUD complications in COVID-19. As we analyzed earlier, SUDs complicate and impair the respiratory system directly, and can intensify the severity of the disease through cardiovascular damage and immune abnormality. There are increasing concerns on capillary endothelial damage or endotheliitis by COVID-19 and SUDs, including the harmful effects on the BBB integrity. If the coronavirus is allowed to migrate across the BBB and to infect the brain, long-term neurological degeneracy is expected, and the treatment will be deemed challenging. SUDs can weaken the immune system, alter and disrupt the HPA axis, and stimulate neuroinflammation with heightened expression of TNFα, IL-1β, and IL-6 in the CNS. Anti-TNFα therapy has been used in severe cases of autoimmune inflammatory disease to control inflammation by downregulating IL-6 and IL-1β [235]. Anti-IL-6 antibody may also be beneficial in inflammation control [236]. These treatments could be helpful for COVID-19 patients with SUDs.

Current antiviral drugs are designed to interfere with viral replication or viral protein processing. For example, the most effective drug, remdesivir, which can shorten the recovery time by 30% [206], inhibits viral RNA replication [212]. Neutralizing antibodies bind to RBD of the S protein, so the virus cannot bind to ACE2. However, clearance of the virus is heavily dependent on the individual’s immune system, including activating phagocytes and natural killer cells. As discussed earlier, several substances of abuse, such as nicotine, marijuana, cocaine, and amphetamines, have shown to suppress immune and antibody responses. Clinical cases indicated that antibody-secreting cells (ASCs), T follicular helper (T_FH_) cells, as well as activated CD4+ and CD8+ T-cells, are critical to symptomatic recovery from COVID-19 [237]. As a result, SUD-induced reduction of T-cell activation will dampen the ability to efficiently clear the virus from the body. For vaccines to work efficiently, robust immune responses are required. CD4+ and CD8+ T-cell responses are essential in protective antiviral immunity by vaccination [238,239]. Due to the immunosuppression and reduction of T-cell responses, individuals with SUDs may not develop sufficient protective antibodies against the virus. A clinical study suggested that SARS-CoV-2 specific immunoglobin G (IgG) antibodies may last only 2-3 months before a steep decline in both asymptomatic and mildly symptomatic COVID-19 patients [240]. Considering their substantially undermined immune systems, the protective antibodies in patients with SUDs are very likely to decline much quicker, making vaccines considerably less effective in these patients. There is a potential risk, although no reported cases yet, that antibodies or vaccines may promote COVID-19 pathogenesis through antibody-dependent enhancement (ADE). While SUDs compromise the immune system, virus-specific antibodies are likely to promote viral entry into various immune cells, including monocytes, macrophages, and B cells [241,242], which may further deteriorate the immune response towards the virus.

COVID-19 pandemic poses tremendous challenges for the treatment of SUDs [243]. The regulatory and policy obstacles for patients with SUDs are intensified in the times of crisis, making it more difficult for healthcare providers to address the needs of SUD patients with the availability of medications. Physical and social distancing requirement renders the face-to-face group treatment and mutual support groups inaccessible. These difficulties will disrupt the treatment of patients with SUDs when these patients start to experience withdrawal symptoms. It has been called for relaxing rules and regulations for these patients to receive treatment, and adopting the model of pharmacy-based addiction care by integrating primary-care and pharmacy prescription, dispense, and management [243].

It is now widely accepted that COVID-19 may stay with us for some extended periods. People with mild or no symptoms can transmit the pathogen as effectively as those with severe symptoms [244,245]. The complications and compromised immune systems associated with SUDs make drug abusers particularly vulnerable to COVID-19. Therapeutics and vaccines currently under development do not address the specific concerns and risk factors for these individuals. There are even greater challenges for people with SUDs under COVID-19 pandemic, as they will experience a higher infectious rate, limited access to healthcare system and support groups, inadequate food and housing, and increased likelihood of homelessness and incarceration. They may also face excessive discrimination, and have higher chances of relapses and overdose death. The research community should heed to the challenges and difficulties these individuals may experience in the pandemic, uncover scientific evidence to link COVID-19 severity and mortality with substance use, and advance effective treatment and prevention strategies for people with SUDs.

## Figures and Tables

**Table 1:** COVID-19 Fatality and Mortality Rates on Comorbid Conditions in China.

Comorbid Conditions	Case Fatality (%)	Mortality (per 100,000 Population)
Overall	2.3	150
None	0.9	50
Hypertension	6.0	380
Diabetes	7.3	450
Cardiovascular diseases	10.5	680
Chronic respiratory diseases	6.3	400
Cancer	5.6	360

* Source: China CDC, http://weekly.chinacdc.cn/en/article/id/e53946e2-c6c4-41e9-9a9b-fea8db1a8f51.

**Table 2:** COVID-19 hospitalization and critical illness on comorbid conditions in New York City.

Comorbid Conditions	Not Hospitalize *N* (%)	Hospitalized *N* (%)	No Critical Illness *N* (%)	Critical Illness *N* (%)
Total cases	2104	1999	932	650
Tobacco use (current or former)	358 (19.5)	520 (26.0)	237 (25.5)	173 (26.6)
Obesity (BMI ≥ 30)	304 (14.4)	796 (39.8)	378 (40.6)	260 (40.0)
Cardiovascular conditions	344 (16.3)	891 (44.6)	391 (42.0)	306 (47.1)
Hypertension	241 (11.5)	742 (37.1)	320 (34.3)	257 (39.5)
Diabetes	111 (5.3)	503 (25.2)	213 (22.9)	176 (27.1)
Asthma or COPD	106 (5.0)	206 (10.3)	91 (9.8)	71 (10.9)

* Source: reference [37].

**Table 3:** Connection of substances of abuse to COVID-19.

Substance	Target System	Pathology	COVID-19
**Tobacco (Nicotine)**	Respiratory system	Main cause of COPD [10,11,82]	Increased severity and mortality [13,81]
	Immune system	Immune suppression, Decreased CD8+ T-cells [83,84]	Higher infection rate [85]
		Increased inflammatory cytokines (TNFα, IL-1β, IL-18) and chemokines (CCL2, CCL8, and CXC3CL1); decreased anti-inflammatory factors, Bcl6, IL-10, and CCL25 [92]	Increased inflammatory cytokines and chemokines, TNFα, IL-1β, IL-6 [61,62,63]
	CNS	BBB leakage through loss of tight junction proteins [86,87,88,89]	Endotheliitis and CNS infection [53,55,56,57]
**Alcohol**	Immune system	Increased proinflammatory cytokines, IL-1β and IL-6, and chemokine CCL-2 [98]	Increased inflammatory cytokines and chemokines, TNFα, IL-1β, IL-6 [61,62,63]
		Spleen atrophy [100].	Impaired production of antibodies and lymphocytes [102]
	CNS	Increased BBB permeability through cytoskeletal and tight junction remodeling [103,104,105].	Endotheliitis and CNS infection [53,55,56,57]
**Marijuana (THC, CBD)**	Respiratory system	Enhanced COPD with tobacco [113,114]	Increased severity and mortality [13,81]
	Immune system	Immunosuppression; reduced antibody response and T lymphocyte activities; reduced migration of macrophage [111]	Increased infection and reduced viral response and clearance [111]
**Opioids (heroine, fentanyl, morphine)**	Respiratory system	Respiratory depression [14,15,119]	Increased severity and mortality [14,15]
	Immune system	Desensitizing HPA axis; inhibiting glucocorticoid release, increased IL-1β; neuroinflammation [120]	Increased opportunistic infections, excessive inflammatory response [117,118,121]
**Cocaine**	Cardiovascular system	Cardiac arrhythmias and acute MI; oxygen imbalance; microvascular diseases and thrombosis [122,123,124,125,126,127,129,130,131,132]	Increased severity and mortality [12,37,38]
	Immune system	Stimulating HPA axis; immunosuppression; defects in antibody formation, lymphocyte proliferation, macrophage and NK activation [141,142]	High incidence of viral infection [142]
	CNS	Increased BBB permeability due to loss of tight junction proteins; rearrangement of cytoskeleton structure [143,144,145,146]	Endotheliitis and CNS infection [53,55,56,57]
**Amphetamine, METH, MDMA**	Cardiovascular system	Hypertension, tachycardia, and cardiomyopathy leading to pulmonary edema [16,163]; Ischemic stroke and hypoxia; restricted blood flow [171,172,173,174]	Increased severity and mortality [12,37,38]
	Immune system	Altered HPA axis, impairing GR and MR expression, immunosuppression [164,165,166,167]	Increased infection rate, depression, anxiety/despair [168,169]
		Increased expression of TNFα, IL-1β, and IL-6; neuroinflammation [185,186,187,188]	Excessive inflammatory response [61,62,63]
	CNS	BBB damage due to loss of tight junction protein; edema [175,176,177,178,179]	Endotheliitis and CNS infection [53,55,56,57,180,181,182]
